
JOURNAL INFORMATION
==============================
NLM Title Abbreviation: Behav Neurol
Journal ID: Behav Neurol
NLM Journal ID: 8914585
Journal ID: BN

Behavioural Neurology

ISSN: 0953-4180
EISSN: 1875-8584
Publisher: Wiley

ARTICLE INFORMATION
==============================
PMCID: PMC5434353
PMID: 21422557
DOI: 10.3233/BEN-2010-0297
Article ID: 320707
Article version: 1
Subjects: Research Article

Multitasking and Prospective Memory: Can Virtual Reality be Useful for Diagnosis?

Banville Frederic 1 *
Nolin Pierre 2
Lalonde Sophie 2
Henry Mylene 2
Dery Marie-Pier 2
Villemure Rene 2
1 University of Quebec at Trois-Rivieres Rehabilitation Center Le Bouclier QC Canada
2 University of Quebec at Trois-Rivieres QC Canada
*Frederic Banville: fredericbanville@videotron.ca
Print publication date: 2010
Electronic publication date: 2011 Mar 7
Volume: 23
Issue: 4
First page: 209
Last page: 211
Received 2011 Jan 31; Accepted 2011 Jan 31
Copyright: Copyright © 2010 Hindawi Publishing Corporation and the authors.
Copyright year: 2010
License: This is an open access article distributed under the Creative Commons Attribution License, which permits unrestricted use, distribution, and reproduction in any medium, provided the original work is properly cited.
License URL: https://creativecommons.org/licenses/by/3.0/

==============================
Behavioural Neurology 23 (2010) 209–211
DOI 10.3233/BEN-2010-0297
IOS Press

### Multitasking and Prospective Memory: Can Virtual Reality be Useful for Diagnosis?

Frederic Banville[a,*], Pierre Nolin[b], Sophie Lalonde[b], Mylene Henry[b], Marie-Pier Dery[b] and Rene Villemure[b]
[a]*University of Quebec at Trois-Rivieres, Rehabilitation Center Le Bouclier, QC, Canada*
[b]*University of Quebec at Trois-Rivieres, QC, Canada*

### 1. Overview

Prospective memory (PM) is defined as the ability to perform an intended action in the future [4]. It is with this type of memory that one can observe the highest number of memory errors made within the context of everyday life [6]. Furthermore, researchers have demonstrated that prospective memory is extremely sensitive to traumatic brain injury (TBI) [5]. In the past few years, research in the field of PM has shown how the frontal lobes are involved in the context of multitasking.

Multitasking is defined by the ability to execute and monitor a set of "goal-oriented" behaviours in order to realize planned actions [3]. Shallice and Burgess [8] have discussed the importance of multitasking as an essential component of prospective memory.

During the last few years, neuropsychological tests have been criticized, especially with regard to everyday functioning. At present, neuropsychological tools sometimes fail to detect subtle and complex deficits in the goal-oriented behaviours in order to realise self-planned actions [8].

We now seem to have at our disposal virtual reality (VR) to compensate for the different limits of traditional assessment. One of the major plusses of VR is its capacity to bring the real world into a laboratory setting thus permitting the control of stimuli and the recording of the patient's answers and behaviours. Over time, some experiments which have used VR technology have demonstrated its ecological validity and its capacity to detect planning or prospective memory deficit [2,7,9].

The objective of the present study is to demonstrate the capacity of VR to detect prospective memory problems.

### 2. Method

### 2.1. Participants

Thirty-one participants (8 women and 23 men) who have sustained a TBI participated in this study. Seven subjects have sustained a moderate TBI and twenty-four a severe TBI. All subjects were evaluated both in a VR condition and a traditional neuropsychological condition. Thirty-one control subjects were matched in gender, education and age with TBI participants.

The mean age for TBI participants was 27 years old (SD = 11 years); they had an average of 12 years of education (SD = 2,5 years). The mean age for control subjects was 27 years (SD = 11 years); they had an average of 12 years of education (SD = 1,61). Statistical analysis has not revealed any differences between the groups in terms of age ($F(1,60) = 2.21$, $p = 0{,}14$) and education ($F(1,60) = 2{,}15$, $p = 0{,}15$).

*Corresponding author: Frederic Banville, Neuropsychologist, P.O. Box 502, Station St-Jerome, St-Jerome, J7Z 5V2, Quebec, Canada. Tel.: +1 450 275 4518; E-mail: fredericbanville@videotron.ca.

ISSN 0953-4180/10/$27.50 © 2010 – IOS Press and the authors. All rights reserved

### *2.2. Material*

This study was conducted with a PC Laptop and an eMagin z800 visor in order to immerse the participants into virtual environments. The virtual environments used to conduct this study were taken from the "Modified Max Payne Environments" developed by Bouchard et al. [1]. The environments in question are the virtual city (which is used in the learning phase) and two virtual apartments (which are used in order to realize the PM assessment).

### *2.3. Procedure*

Three virtual prospective memory tasks are done while visiting two apartments. The subject must: 1) feed fish at 11:41, 2) turn off the fan when leaving the bedroom of the larger apartment, and 3) take the lease from the counter after visiting the smaller apartment. Two traditional prospective memory tasks have been realized; they were analogous to those found in the Rivermead Behavioural Memory Test (RBMT) which is frequently used in prospective memory research. More specifically, the participant has to: 1) ask the evaluator to give him a business card and 2) ask the evaluator to give back to him a personal object. Finally, the ongoing task has consisted in visiting two apartments with the aim of renting one. The participant was voice-recorded during the visit and had to describe what he/she saw in terms of his/her preference in living in one of the two apartments.

### *2.4. Results*

Three scores were taken from the VR condition: precision in the realization of prospective task; time taken to make the visit; and the success (or not) of virtual PM tasks. One score in fact, comes from the traditional prospective memory evaluation: it is a measurement of success (or not) in performing PM tasks. We used MANOVA for our first statistical analysis. The analysis of the principal effect has shown a significant group difference (F(1,50) $=$ 8,79, $p <$ 0,01) in the four dependant variables. More specifically, the simple effects did not show a significant group difference in the success of PM tasks both in VR (F(1,50) $=$ 1,25, $p >$ 0,05) or traditional tasks (F(1,50) $=$ 2,73, $p >$ 0,05). However, and more interestingly, the TBI group was less precise (F(1,50) $=$ 5,70, $p <$ 0,05) and took significantly more time to complete the task (F(1,50) $=$ 7,57, $p <$ 0,01) in comparison with the control group. Using the same dependant variables, a discriminant analysis has correctly classified 71% of the participants in the correct group (TBI or control).

### 3. Discussion

The principal aim of this experiment was to demonstrate the utility of VR technology in order to detect prospective memory problems after traumatic brain injury. The results here have demonstrated, as elsewhere [9], that the realization of delayed intention for both experimental and control groups was normal. Nevertheless, the TBI participants' executive dysfunctions can be detected in the way they had difficulties in managing well the interference and cognitive overload generated by the multitasking condition. In fact, TBI participants took more time to realize virtual prospective memory tasks; they made more errors in terms of quality of realization; and they were less structured in the task than control group participants.

These research results seem to suggest that in order to obtain a good measurement of prospective memory, one must include multitasking conditions and several quantitative and qualitative measurements. This way, we can detect subtle behaviours which indicate a malfunctioning of cognitive management especially with regard to the executive component in the realisation of the intended action. This experiment, like previous tests, is a testament to the usefulness of virtual reality, which can classify correctly the majority of participants as TBI patients or control agents. Moreover, it appears that virtual reality protocol can be used as a complement with traditional neuropsychological tools in order to assess PM after traumatic brain injury. This is especially the case when we have to detect subtle everyday problems in TBI patients. In the end, more work is needed and more data collection is required before we can come to a definite conclusion.

### References

[1] S. Bouchard, S. Côté and S.C.D. Richard, Virtual reality applications for exposure. In *Handbook of Exposures Therapy*, D.C.S. Richard and D. Lauterbach, eds, Chapter 16, 2007, pp. 347–388.

[2] B.M. Brooks, F.D. Rose, J. Potter, S. Jayawardena and A. Morling, Assessing stroke patients' prospective memory using virtual reality, *Brain Injury* **18**(4) (april) (2004), 391–401.

[3] P.W. Burgess, I. Dumontel, S.J. Gilbert, J. Okuda, M.L. Schölvinick and J.S. Simons, On the role of rostral prefrontal cortex (Aera 10) in prospective memory. In: *Prospective Memory: Cognitive, Neuroscience, Developmental and Applied Perspective*, M. Kliegel, M.A. McDaniel and G.O. Einstein eds, Lawrence Erlbaum Associates, 2008, pp. 235–260.

[4] J. Ellis, Prospective memory or the realisation of delayed intentions: a conceptual framework for research. In: *Prospective Memory: Theory and Applications*, M. Brandimonte, G.O. Einstein and M.A., McDaniels, eds, Hillsdale, NJ: Lawrence Erlbaum Associates, 1996, pp. 1–22.

[5] J.M. Fleming, D. Shum, J. Strong and S. Lightbody, Prospective memory rehabilitation for adults with traumatic brain injury: A compensatory training program, *Brain Injury* **19**(1) (2005), 1–10.

[6] M. Kliegel and M. Martin, Prospective memory research: Why is it relevant? *International Journal of Psychology* **38** (2003), 193–194.

[7] P. McGeorge, L.H. Phillips, J.R. Crawford, S.E. Garden, S. Della Sala, A.B. Milne et al., Using virtual environments in the assessment of executive dysfunction, *Presence* **10** (2001), 375–383.

[8] T. Shallice and P.W. Burgess, Deficits in strategy application following frontal lobe damage in man, *Brain* **114** (1991), 727–741.

[9] S. Sweeney, D. Kersel, R.G. Morris, T. Manly and J.J. Evans, The sensitivity of a virtual reality task to planning and prospective memory impairments: Group differences and the efficacy of periodic alerts on performance, *Neuropsychological Rehabilitation* **20**(2) (2010), 239–263.
